# Molecular characterization of hybrid virulence plasmids in ST11-KL64 KPC-2-producing multidrug-resistant hypervirulent *Klebsiella pneumoniae* from China

**DOI:** 10.3389/fmicb.2024.1353849

**Published:** 2024-03-14

**Authors:** Fushan Zhang, Leyuan Li, Yuxin Zhao, Huiyue Dong, Buhui Zhao, Xiaoyu Zhao, Ziwei Xia, Leizi Chi, Yan Wang, Ruichao Li, Shangshang Qin, Xiangjing Fu

**Affiliations:** ^1^Department of Clinical Laboratory, The First Affiliated Hospital of Zhengzhou University, Zhengzhou, Henan, China; ^2^School of Pharmaceutical Sciences, Zhengzhou University, Zhengzhou, Henan, China; ^3^Key Laboratory of Advanced Drug Preparation Technologies, Ministry of Education, Zhengzhou University, Zhengzhou, Henan, China; ^4^Jiangsu Co-Innovation Center for Prevention and Control of Important Animal Infectious Diseases and Zoonoses, College of Veterinary Medicine, Yangzhou University, Yangzhou, Jiangsu, China

**Keywords:** *bla*_KPC−2_-bearing virulence plasmid, MDR-virulence plasmid, hypervirulent, ST11-KL64, CRKP

## Abstract

**Introduction:**

Carbapenem-resistant hypervirulent *Klebsiella pneumoniae* (CR-HvKP) strains combining virulence and multidrug resistance (MDR) features pose a great public health concern. The aim of this study is to explore the evolutionary characteristics of virulence in CR-HvKP by investigating the genetic features of resistance and virulence hybrid plasmids.

**Methods:**

The resistance and virulence phenotypes were determined by using antimicrobial susceptibility testing and the mouse bacteremia infection model, respectively. Plasmid profiles were investigated by S1 nuclease pulsed-field gel electrophoresis (S1-PFGE) and Southern blotting, conjugation assay, and whole genome sequencing (WGS). Bioinformatics tools were used to uncover the genetic features of the resistance and virulence hybrid plasmids.

**Results:**

Two ST11-KL64 CRKP clinical isolates (KP18-3-8 and KP18-2079), which exhibited enhanced virulence compared with the classic CRKP, were detected positive for *bla*_KPC−2_ and *rmpA2*. The virulence level of the hypermucoviscous strain KP18-3-8 was higher than that of KP18-2079. S1-PFGE, Southern hybridization and WGS analysis identified two novel hybrid virulence plasmids in KP18-3-8 (pKP1838-KPC-vir, 228,158 bp) and KP18-2079 (pKP1838-KPC-vir, 182,326 bp), respectively. The IncHI1B/repB-type plasmid pKP1838-KPC-vir co-harboring *bla*_KPC−2_ and virulence genes (*rmpA2, iucABCD* and *iut*A) but lacking type IV secretion system could transfer into non-hypervirulent ST11 *K. pneumoniae* with the assistance of a helper plasmid in conjugation. The IncFII/IncR-type virulence plasmid pKP18-2079-vir may have been generated as a result of recombination between a typical pLVPK-like virulence plasmid and an MDR plasmid.

**Conclusion:**

Our studies further highlight co-evolution of the virulence and resistance plasmids in ST11-CRKP isolates. Close surveillance of such hybrid virulence plasmids in clinical *K. pneumoniae* should be performed.

## Introduction

As the most predominant and prevalent species within carbapenem-resistant *Enterobacterales* (CRE), carbapenem-resistant *Klebsiella pneumoniae* (CRKP) is notable for its considerable resistance and the treatment challenges. Current studies revealed that multidrug-resistant (MDR) or extensive drug-resistant (XDR) CRKP isolates were only susceptible to tigecycline, polymyxin, and new β-lactamase inhibitor compound preparations such as ceftazidime/avibactam (Yang et al., 2022), and these agents were also prescribed for the treatment of CRKP infections in China (Yahav et al., 2020; Han et al., 2022).

Recently, the emergence and increasing reports of carbapenem-resistant hypervirulent *K. pneumoniae* (CR-HvKP), which are simultaneously hypervirulent and multidrug resistant, represent a new serious threat in China for clinical treatment and therefore be recognized as a real superbug (Gu et al., 2018). The occurrence of CR-HvKP could be through either horizontal transfer of resistance plasmids into HvKP strains or through acquisition of the virulence plasmids in CRKP strains (Yang et al., 2021; Han et al., 2022), and the latter pathway was more frequently reported to be responsible for the emergence of CR-HvKP isolates since the MDR *K. pneumoniae* isolates have demonstrated a greater tendency to acquire virulence genes than hypervirulent clones to acquire resistance genes (Wyres et al., 2019). A recent national surveillance concerning CRKP from China identified a high proportion of CRKP isolates (34.2%) co-harboring *bla*_KPC−2_ and virulence plasmids in China (Wang et al., 2018; Zhang et al., 2020). The co-existence of virulence and MDR plasmids in the same CR-HvKP strain provide the opportunity for recombination between the two types of plasmids. Our previous report described the fusion of a virulence plasmid p17-16-vir (**MK191024**) and an MDR plasmid p17-16-CTX (**MK192097**) during conjugation (Li et al., 2020), and recent studies also reported the fusion of the non-conjugative virulence plasmid with the *bla*_KPC_-positive plasmid through homologous recombination (Xie et al., 2021; Zhao et al., 2022). In addition, recent research studies revealed that the pLVPK-like virulence plasmid can be transferred from HvKP strains to ST11 CRKP and *Escherichia coli* strains via a self-transferable IncF plasmid in a variety of modes (Li et al., 2020; Xu et al., 2021). Furthermore, IS element-mediated insertion of *bla*_KPC−2_ into the virulence plasmid is another way to generate hybrid plasmids that simultaneously expressed the carbapenem resistance- and hypervirulence-associated phenotypes (Dong et al., 2018; Jin et al., 2021). The emergence of such plasmids, coupled with the co-transfer of resistance and virulence determinants, may directly facilitate the transformation of classical *K. pneumoniae* into CR-HvKP. This phenomenon further exacerbates the spread of CR-HvKP strains, posing a severe threat to public health and requiring continuous monitoring. In this study, we have reported two novel hybrid plasmids encoding MDR/carbapenem resistance and virulence phenotypes in KPC-2 producing *K. pneumoniae* belonging to ST11-KL64, which is regarded as the most commonly detected type responsible for the majority of CRKP infections in China (Zhang et al., 2020; Wang et al., 2022), indicating further evolution of the pLVPK-like virulence plasmids in CRKP.

## Materials and methods

### Bacterial isolates, antimicrobial susceptibility testing, and PCR detection

Clinical *K. pneumoniae* strain 18-3-8 (KP18-3-8) was obtained from the urine culture of a 75-year-old man hospitalized in a respiratory unit of a local hospital in Pingdingshan city of Henan province on 14 July 2018, and KP18-2079 was recovered from the blood culture of a 42-year-old man, who was diagnosed with epilepsy and hospitalized at a neurosurgery unit of a teaching hospital of Zhengzhou University on 22 November 2018. The minimum inhibitory concentrations (MICs) of antimicrobial agents commonly used for the treatment of *Enterobacterales* infections were determined by the broth microdilution method according to the Clinical and Laboratory Standards Institute (CLSI) guidelines (CLSI, 2018). The MIC results of the tested antibiotics were interpreted using the CLSI breakpoints, except tigecycline, which was interpreted in accordance with the EUCAST breakpoints (http://www.eucast.org/clinical_breakpoints/). An *E. coli* strain ATCC 25922 was used as the quality control. PCR and sequencing methods were employed to detect the presence of carbapenemase-encoding genes (*bla*_KPC_, *bla*_NDM_, *bla*_IMP_, and *bla*_OXA−48−like_) and virulence factors *rmpA/A2* in CRKP isolates (Qin et al., 2014; Yu et al., 2018).

### S1-PFGE and Southern blotting

S1-PFGE and Southern blotting were performed according to the method described in our previous study (Li et al., 2020). In brief, the agarose gel plugs containing whole-cell DNA of the strain were treated with S1 nuclease (TaKaRa, Dalian, China) and then PFGE was performed under the following conditions: 1% agarose solution for 18 h at 6 V/cm and 14°C, with a pulse angle of 120° and the pulse time linearly ramped from 2.16 s to 63.8 s. Digoxigenin-labeled *bla*_KPC−2_ and *rmpA2*-specific probes were hybridized with linear plasmids separated by S1-PFGE on nylon membranes (Millipore, USA) and then detected using an NBT/BCIP color detection kit (Roche, Germany).

### Conjugation experiments

The transferability of hybrid plasmids was investigated by conducting conjugation experiments. KP18-3-8 and KP18-2079 served as the donors, with hygromycin-resistant ST11 *K. pneumoniae* HS11286YZ6 as the recipient strain (Xie et al., 2018). Donor and recipient strains, grown to logarithmic growth phase (OD_600_ of 0.6–0.8), were inoculated into Luria Bertani (LB) broth, were mixed at a ratio of 1:1 between donor and recipient strains, and were incubated for 24 h at 30°C. Transconjugants were selected on LB agar plates supplemented with 2 μg/mL meropenem or 2 μg/mL potassium tellurite (K_2_TeO_3_) plus 200 μg/mL hygromycin and identified by PCR targeting *bla*_KPC−2_ or *rmpA2*.

### Evaluation of hypermucoviscous and virulence phenotypes

The hypermucoviscous phenotype was determined using the string test (Russo et al., 2018). The mouse bacteremia infection model was used to assess the virulence of various *K. pneumoniae* strains as described previously (Yang et al., 2019). The non-hypermucoviscous ST1-K19 *K. pneumoniae* strain, KP18-208, was used as the control for low virulence (Qin et al., 2020), while an ST268-K20 *K. penumoniae* strain, KP19-2065, served as the control for hypervirulence. Mice in each group were infected intravenously with 5.0 × 10^5^ CFU and 5.0 × 10^6^ CFU of each *K. pneumoniae* strain tested. The survival rate of the mice was recorded daily for 5 days. The survival curves were plotted using the Kaplan-Meier method, and the statistical analysis was performed using the Log-rank test in GraphPad 9.5.1.

### Whole genome sequencing and analysis

Genome DNA of the two clinical strains was extracted using the Wizard Genomic DNA Purification Kit (Promega, Madison, WI), quantified using Nanodrop and Qubit fluorometer, and sequenced via Illumina paired-end 2×150 bp platform. Nanopore sequencing long-read DNA libraries were constructed using a rapid sequencing kit (SQK-RAD004, ONT, Oxford, UK) and a rapid barcode kit (SQK-RAD004, ONT, Oxford, UK) according to the manufacturer's protocol. Sequencing was performed using MinION (ONT, Oxford, UK). Different bioinformatics tools including Unicycler and Flye were utilized to assemble the complete genome sequences (Wick et al., 2017; Kolmogorov et al., 2019). The RAST tool was used to annotate the genomes, and the sequences were further modified manually (https://rast.nmpdr.org/). Different databases and tools, including Resfinder and PlasmidFinder (https://cge.cbs.dtu.dk/services/) from the Center for Genomic Epidemiology (CGE) and ISFinder (https://www-is.biotoul.fr/), were used to perform genomic analysis and visualization. Easyfig (https://github.com/mjsull/Easyfig) and BRIG (http://sourceforge.net/projects/brig/) were used for displaying plasmid comparison maps (Alikhan et al., 2011; Sullivan et al., 2011).

## Results and discussion

### Resistance phenotype and genotype of KPC-2-producing ST11-KL64 *K. pneumoniae* isolates

**Figure 1:**
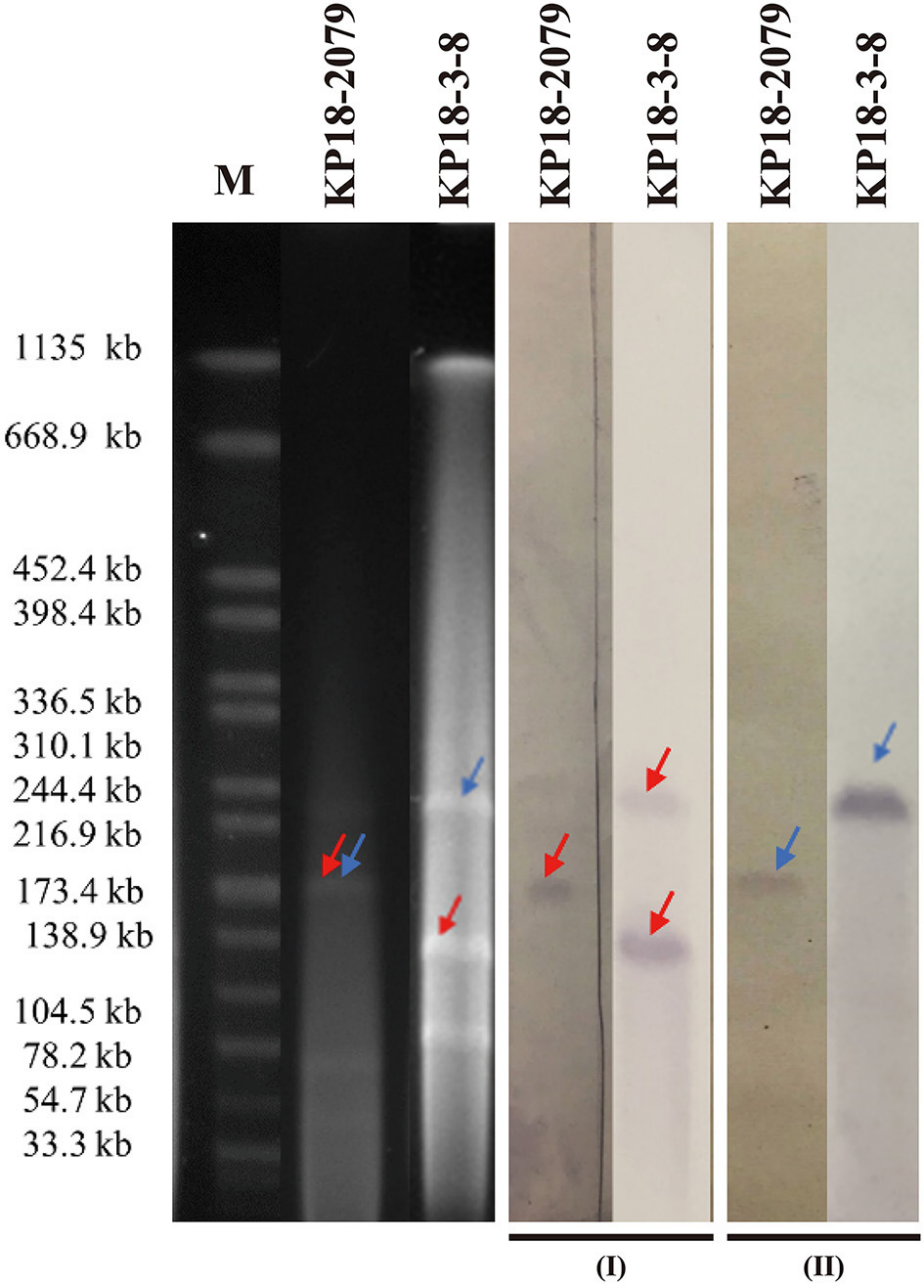
S1-PFGE and Southern hybridization analyses of KP18-2079 and KP18-3-8 using the *bla*_KPC−2_ (I) and *rmpA2* probes (II). Lane M, reference standard strain H9812 restricted with *XbaI* (sizes are given in kilobases).

Both isolates KP18-3-8 and KP18-2079 were resistant to carbapenem and cephalosporins but were susceptible to colistin (Table 1). PCR-based screening for carbapenemase and virulence-encoding genes identified both *bla*_KPC−2_ and *rmpA2* genes in each isolate. Further analysis using S1-PFGE and Southern blotting techniques with *bla*_KPC−2_ and *rmpA2*-specific probes indicated that these two genes were localized on plasmids of the same size in both KP18-3-8 (approximately 220 kb) and KP18-2079 (approximately 180 kb; Figure 1) strains, and therefore, we speculated that the two genes were located on the same plasmid.

**Table 1:** Antimicrobial susceptibility testing of KP18-3-8, KP18-2079, and the transconjugant of KP18-3-8.

**Strain**	**MIC (**μ**g/mL)**^**a**^												
	**MEM**	**GEN**	**ATM**	**CAZ**	**CIP**	**CHL**	**DOX**	**TGC**	**AMK**	**CST**	**AMP**	**CFZ**	**TZP**
**Clinical isolate**													
KP18-3-8	64	>128	>64	>64	>8	>128	64	4	>128	0.25	>256	>256	>1,024
KP18-2079	64	>128	>64	>64	>8	64	128	2	>128	0.5	>256	>256	>512
**Recipient**													
HS11286YZ6	≤ 0.06	0.5	32	4	4	4	4	1	0.5	1	>256	>256	>512
**Transconjugant**													
YZ6/pKP18-3-8-KPC-vir^b^	4	4	64	16	8	8	2	0.5	1	1	>256	>256	>1,024

a MEM, meropenem; GEN, gentamicin; ATM, aztreonam; CAZ, ceftazidime; CIP, ciprofloxacin; CHL, chloramphenicol; DOX, doxycycline; TGC, tigecycline; AMK, amikacin; CST, colistin; AMP, ampicillin; CFZ, cefazolin; TZP, piperacillin-tazobactam.

b YZ6/pKP18-3-8-KPC-vir: KPC-positive transconjugant of *K. pneumoniae* HS11286YZ6, selected by using 200 μg/mL hygromycin and 2 μg/mL meropenem.

To uncover the genetic basis of the resistance and virulence in the two strains, whole-genome sequences of KP18-3-8 and KP18-2079 were obtained using a combination of the Illumina (short-read) and Nanopore MinION (long-read) sequencing platforms. KP18-3-8 belonged to ST11 and serotype KL64 and contained one chromosome of ~5.54 Mbp and five plasmids, namely pKP1838-KPC-vir (**MT035874**), pKP1838-KPC (**MT232812**), pKP1838-IncFII (**MT035876**), pKP1838-Col (**MT035877**), and pKP1838-5kb (**MT035878**) of 228,158, 130,717, 87,095, 10,060, and 5,596 bp in size, respectively. In addition, KP18-2079, which was also a ST11-KL64 strain, contained a chromosome of ~5.51 Mbp and harbored six plasmids, namely pKP18-2079-vir (**MT090958**), pKP18-2079-KPC (**MT090959**), pKP18-2079-tetA (**MT090960**), pKP18-2079-54kb (**MT090961**), pKP18-2079-11kb (**MT090962**), and pKP18-2079-5kb (**MT090963**), with sizes of 182,326, 186,564, 84,699, 54,874, 11,970, and 5,596 bp, respectively. Resistome and virulence genes of KP18-3-8 and KP18-2079 are summarized in Table 2. Interestingly, along with the results of S1-PFGE and Southern blotting, we found that KP18-3-8 carried two *bla*_KPC−2_-bearing plasmids, namely pKP1838-KPC-vir and p1838-KPC, and the co-occurrence of *bla*_KPC−2_ and *rmpA2* was observed in pKP1838-KPC-vir. Meanwhile, the *bla*_KPC−2_ and *rmpA2* genes in KP18-2079 were carried by separated plasmids (pKP18-2079-vir and pKP18-2079-KPC) with comparable size.

**Table 2:** Molecular characteristics of XDR CRKP strains KP18-3-8, KP18-2079 and their plasmids.

**Isolate**	**MLST-serotypes**	**Chromosome or plasmid/size**	**NCBI accession no**.	**Plasmid type**	**Resistance genes**	**Virulence genes**
KP18-3-8	ST11-KL64	Chromosome (5,547,989 bp)	CP048430	–	*bla*_SHV−11_ *, sul1, fosA, aadA2b*	*fimABCDEFGHIK, irp1, irp2, mrkABCDFHIJ, ybtAEPQSTUX, entABCDEFS, fepABCDG*
		pKP1838-KPC-vir (228,158 bp)	MT035874	IncHI1B(pNDM-MAR)/repB	*bla*_KPC−2_	*rmpA2, iucABCDiutA, rmpA, peg344, iroNE*
		pKP1838-KPC (130,717 bp)	MT232812	IncFII(pHN7A8)/IncR	*bla*_KPC−2_, *bla*_TEM−1B_, *rmtB*1, *bla*_SHV−11_	
		pKP1838-IncFII (87,095 bp)	MT035876	IncFII	*catA*2, *qnrS*1, *tet*(*A*), *dfrA*14, *sul*2, *bla*_LAP−2_	
		pKP1838-Col (10, 060 bp)	MT035877	ColRNAI		
		pKP1838-5kb (5,596 bp)	MT035878	Untypable		
KP18-2079	ST11-KL64	Chromosome (5,518,817 bp)	CP048933	–	*bla*_SHV−11_ *, sul1, fosA, aadA2b*	*fimABCDEFGHIK, irp1, irp2, mrkABCDFHIJ, ybtAEPQSTUX, entABCDEFS, fepABCDG*
		pKP18-2079-vir (182,326 bp)	MT090958	IncFII(pHN7A8)/IncR	*bla*_TEM−1B_, *bla*_CTX−M−65_, *fosA_3_, rmtB1*	*rmpA2, iucABCDiutA, rmpA, peg344, iroNE*
		pKP18-2079-KPC (186,564 bp)	MT090959	IncHI1B(pNDM-MAR)/repB	*bla*_KPC−2_, *bla*_SHV−12_	
		pKP18-2079-tetA (84,699 bp)	MT090960	Untypable	*tet*(A), *qnrS*1, *sul*2, *dfrA*14, *bla*_LAP−2_	
		pKP18-2079-54kb (54,881 bp)	MT090961	Untypable		
		pKP18-2079-11kb (11,970 bp)	MT090962	ColRNAI		
		pKP18-2079-5kb (5,596 bp)	MT090963	Untypable		

### Identification of an IncHI1B/repB-type *bla*_kpc−2_-bearing virulence plasmid

**Figure 2:**
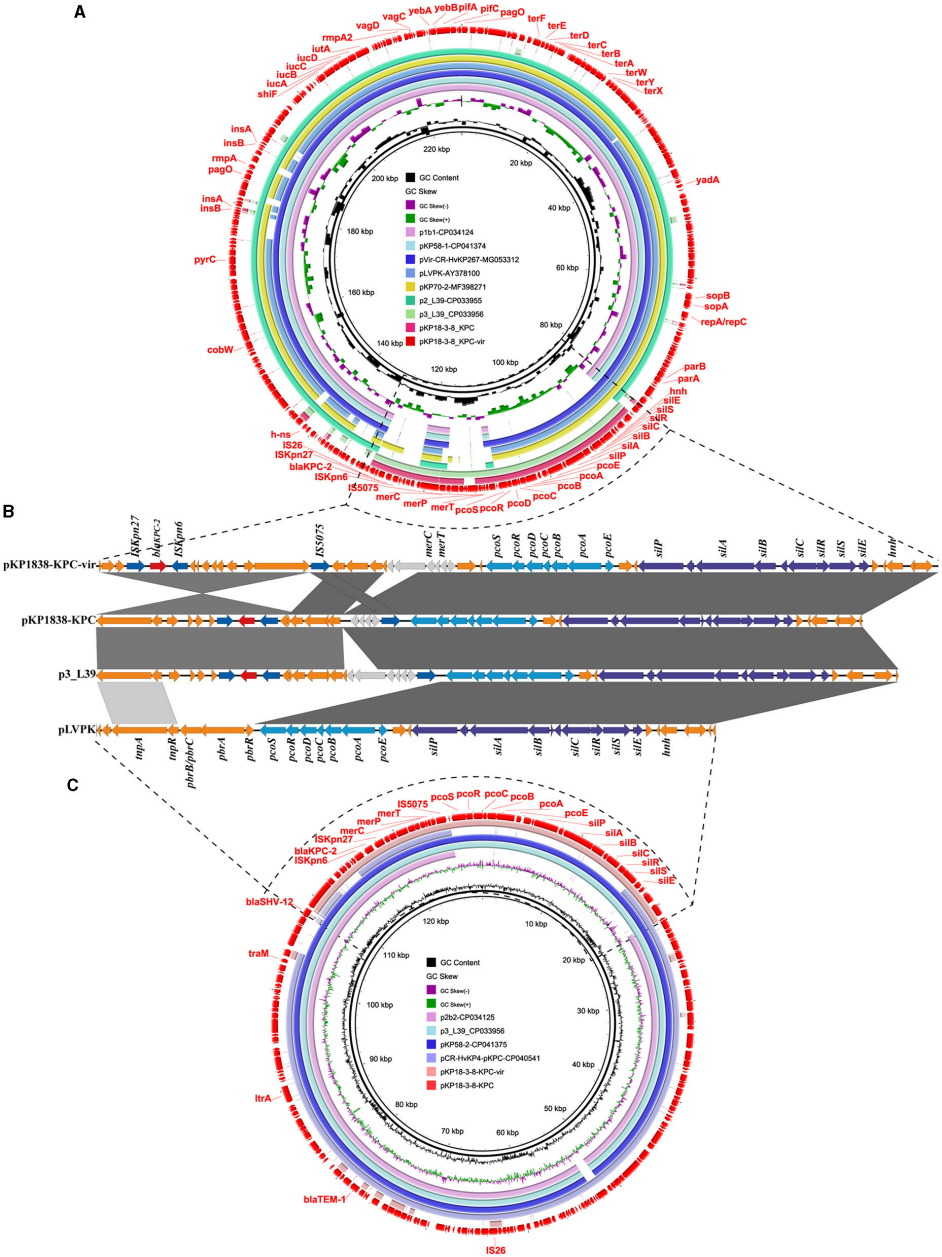
The *rmpA2* and *bla*_KPC−2_ co-harboring plasmid pKP1838-KPC-vir and the *bla*_KPC−2_-bearing plasmid pKP1838-KPC in *Klebsiella pneumoniae* strain KP18-3-8. Circular and linear comparisons of pKP1838-KPC-vir, pKP1838-KPC, p3_L39, and pLVPK plasmids. The pKP1838-KPC-vir and pKP1838-KPC plasmids located at the outermost circle was used as the reference plasmid to perform sequence alignment with BLASTn by BRIG software.

An IncHI1B/repB-type plasmid pKP1838-KPC-vir (**MT035874**) with a length of 228,158 bp was found to be the largest plasmid in the CRKP strain, KP18-3-8. It shared 99% of its identity and 93% of its coverage with a typical non-conjugative pLVPK-like virulence plasmid pvir-CR-HvKP267 (**MG053312**), which was carried by a tigecycline resistant KPC-2 producing hypervirulent *K. pneumoniae* KP267 isolated in 2015 in our previous study (Yao et al., 2018). Notably, compared with pvir-CR-HvKP267, the plasmid pKP1838-KPC-vir carried an additional 19 kb segment containing the carbapenemase-encoding *bla*_KPC−2_ gene, indicating that pKP1838-KPC-vir might have undergone further evolution by integrating exogenous drug resistance genes on the backbone of traditional virulence plasmids. In the KP18-3-8 strain, two *bla*_KPC−2_ genes were identified, each borne on distinct plasmids, pKP1838-KPC-vir and pKP1838-KPC. Detailed comparative analyses of the gene surrounding *bla*_KPC−2_ revealed a 45-kb segment harboring the gene within a non-Tn4401 element (NTE_KPC−1b_), along with gene clusters conferring resistance to heavy metals such as mercury, copper, and silver. Despite a high degree of sequence homology (99% query coverage and 100% nucleotide identity between the segments), notable variations were observed in their genomic arrangement, including an inversion of the entire NTE_KPC−1b_ element associated with the IS*26* element in pKP1838-KPC relative to pKP1838-KPC-vir (Figure 2). Based on the above observations, we supposed that the plasmid pKP1838-KPC-vir co-harboring *bla*_KPC−2_ and virulence factors possibly evolved from a typical pLVPK-like virulence plasmid, and the genetic environment surrounding *bla*_KPC−2_ on pKP1838-KPC-vir similar to that on the *bla*_KPC−2_-bearing plasmid pKP1838-KPC indicates that pKP1838-KPC might have been the source of the *bla*_KPC−2_-carrying fragment for the plasmid pKP1838-KPC-vir.

The conjugative assay revealed that, despite lacking a type IV secretion system (T4SS), pKP1838-KPC-vir was successfully transferred to *K. pneumoniae* HS11286YZ6 at a frequency of 6.37 × 10^−8^ per donor cell. Our previous study and recent reports demonstrated that the non-conjugative virulence plasmids could be mobilized by the conjugative plasmids belonging to IncF (Li et al., 2020; Xu et al., 2021). Thus, we suggested that the plasmid pKP1838-IncFII, which contained four necessary components [*oriT*, relaxase gene, type IV coupling protein (T4CP) gene, and T4SS gene cluster] for self-transfer in the same strain, might have served as a helper plasmid by sharing its T4SS with pKP1838-KPC-vir to transfer.

A recent nationwide surveillance study on CRKP in China revealed that over 90% CRKP isolates were KPC-type carbapenemase producers, especially KPC-2, and over 30% KPC-2 producing CRKP isolates were positive for one or more plasmid-borne virulence genes such as *rmpA, rmpA2, iucA*, and *iroN* (Zhang et al., 2020). Typically, the *bla*_KPC−2_ and virulence genes were carried by distinct plasmids. However, two recent reports described the homologous recombination-mediated fusion of the KPC plasmid and the virulence plasmid during the conjugation process, resulting in the generation of conjugative hybrid plasmids (Xie et al., 2021; Zhao et al., 2022). In addition to this homologous recombination pathway to generate the hybrid *bla*_KPC−2_-bearing virulence plasmids, *bla*_KPC−2_ was rarely reported inserted into the virulence plasmid backbone. To date, only two plasmids, namely pKP70-2 (**MF398271**) and pCRHV-C2244 (**MT644086**), which were presumed to be generated as a consequence of an IS*26*-mediated *bla*_KPC_*-*bearing fragment inserted into the virulence plasmid, were identified in the ST23-K1 HvKP strain KP70-2 isolated from China and the ST11-K64 CRKP strain C2244 isolated from China, respectively (Dong et al., 2018; Jin et al., 2021). However, there was no additional *bla*_KPC−2_-bearing plasmid other than the hybrid virulence- and resistance-encoding plasmid in each strain. In this study, the co-occurrence of *bla*_KPC−2_ that was carried by the NTE_KPC−1b_ element in both virulence and MDR plasmids in the same isolate provided a more direct evidence of recombination events that occurred between virulence and MDR plasmids.

### Characteristics of an IncFII/IncR type multi-drug resistant-virulence plasmid

**Figure 3:**
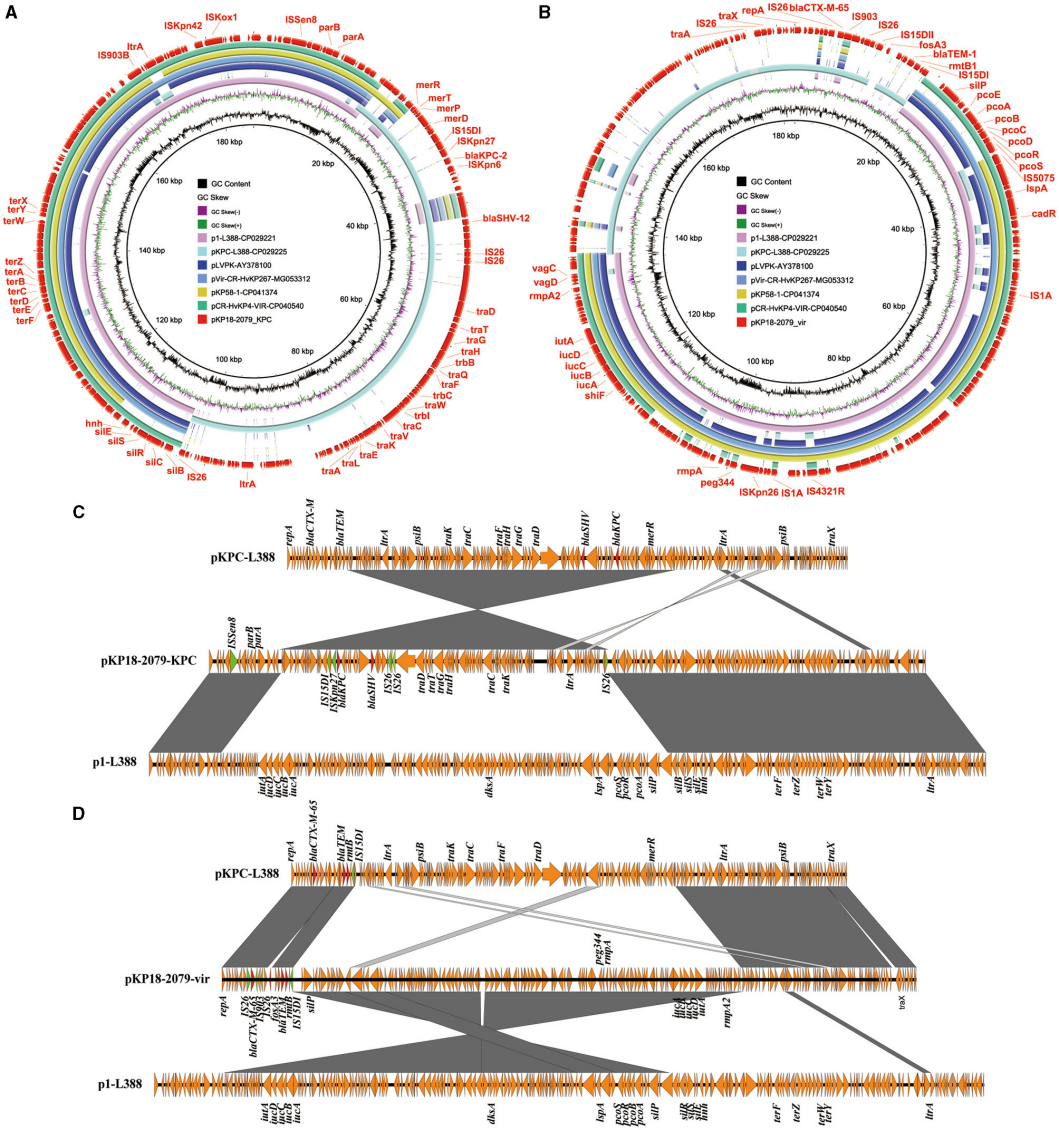
The MDR virulence plasmid p18-2079-vir and the *bla*_KPC−2_-bearing plasmid p18-2079-KPC in *Klebsiella pneumoniae* strain KP18-2079. **(A, B)** Circular maps of plasmid p18-2079-KPC and p18-2079-vir recovered from *K. pneumoniae* KP18-2079 with plasmids recorded in the NCBI database by BLAST Ring Image Generator (BRIG). **(C, D)** Linear alignment of plasmid p18-2079-KPC and p18-2079-vir recovered from *K. pneumoniae* KP18-2079 using Easyfig.

An 182,326 bp IncFII/IncR-type virulence plasmid pKP18-2079-vir (**MT090958**) containing 237 open reading frames, with GC contents of 50.60%, was identified in *K. pneumoniae* KP18-2079 (Table 2). This hybrid plasmid harbored both virulence genes (*iutA*-*iucABCD, rmpA, rmpA2*, and *peg344*) and multiple antimicrobial resistance determinants, including beta-lactamase genes *bla*_TEM−1B_ and *bla*_CTX−M−65_, aminoglycoside resistance gene *rmtB1*, and phosphonic acids resistance gene *fosA3*, simultaneously. The BLAST analysis showed that this type of plasmid has not been reported previously, and it shared 99% identity and 67% coverage with the virulence plasmid p1-L388 (**CP029221**), a typical pLVPK-like virulence plasmid carrying no resistance genes in a ST11 KPC-2-producing *K. pneumoniae* clinical isolate L388 (Figures 3A, C). The comparative genomic analysis further revealed that pKP18-2079-vir contained two genetically and physically distinct modules: an ~117 kb reversed p1-L388-derived module harboring virulence genes (*iut*A-*iuc*ABCD, *rmp*A, *rmp*A2, and *peg*344) and copper resistance genes (*pcoEABCDRS*) and an ~65 kb module carrying a multidrug resistance region that was essentially homologous to pKPC-L388 (**CP029225**), a common *bla*_KPC−2_-bearing IncFII plasmid other than p1-L388 in *K. pneumoniae* L388. The *fosA3* gene encompassed by two IS*26* elements was absent in pKPC-L388 (Figures 3A, C). Interestingly, we further found that the remaining parts of p1-L388 and pKPC-L388 were present in the plasmid pKP18-2079-KPC (**MT090959**) and formed a novel IncHI1B/IncF_repB_-type *bla*_KPC−2_-bearing plasmid in *K. pneumoniae* KP18-2079 (Figures 3B, D). These observations indicated that pKP18-2079-vir (182,326 bp) and p18-2079-KPC (186,564 bp) harbored by KP18-2079 might be generated as a consequence of genetic rearrangement events between p1-L388 (217,870 bp) and pKPC-L388-like (145,851 bp) plasmids.

### Virulence of KPC-2-producing ST11-KL64 *K. pneumoniae* isolates

**Figure 4:**
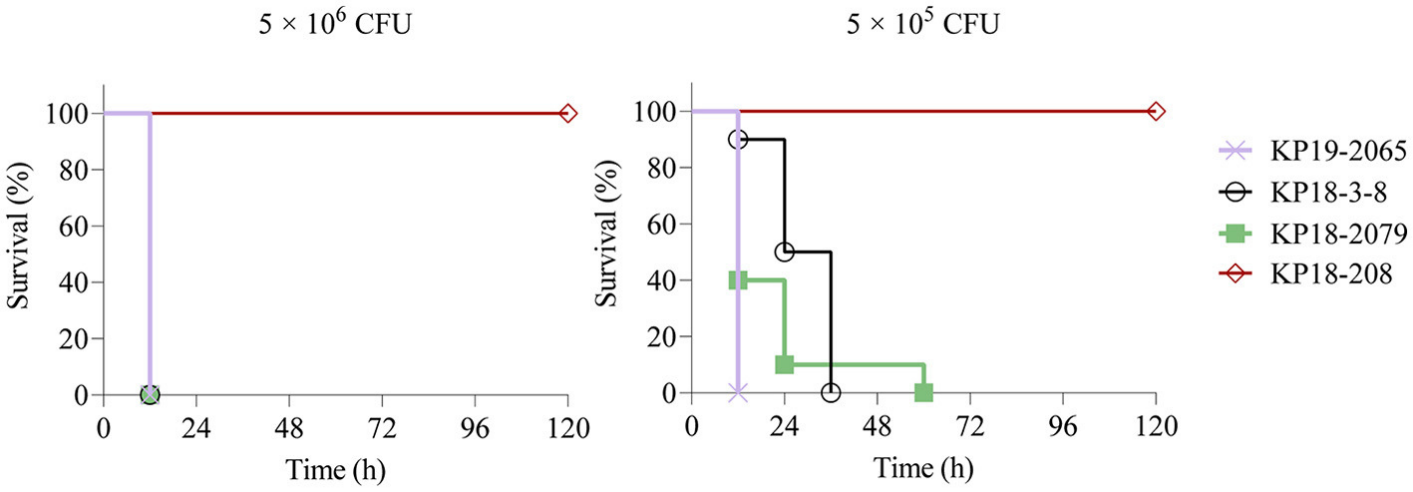
Virulence potential of *Klebsiella pneumoniae* KP18-3-8 and KP18-2079 in a mouse infection model with an inoculum of 5 × 10^6^ CFU and 5 × 10^5^ CFU. Survival of mice (*n* = 10) infected by the indicated inoculum of each *K. pneumoniae* strain at 120 h is shown. The test strains included *K. pneumoniae* strain KP18-3-8, *K. pneumoniae* strain KP18-2079, ST268-K20 hypervirulent *K. pneumoniae* strain KP19-2065 (hypervirulence control), and ST1-K19 classic *K. pneumoniae* strain KP18-208 (low-virulence control). A log-rank (Mantel-Cox) test was performed for the indicated curves. A significant difference (*P* < 0.0001) was observed between KP18-3-8/ KP18-2079 and KP18-208 in 5 × 10^6^ CFU and 5 × 10^5^ CFU.

**Figure 5:**
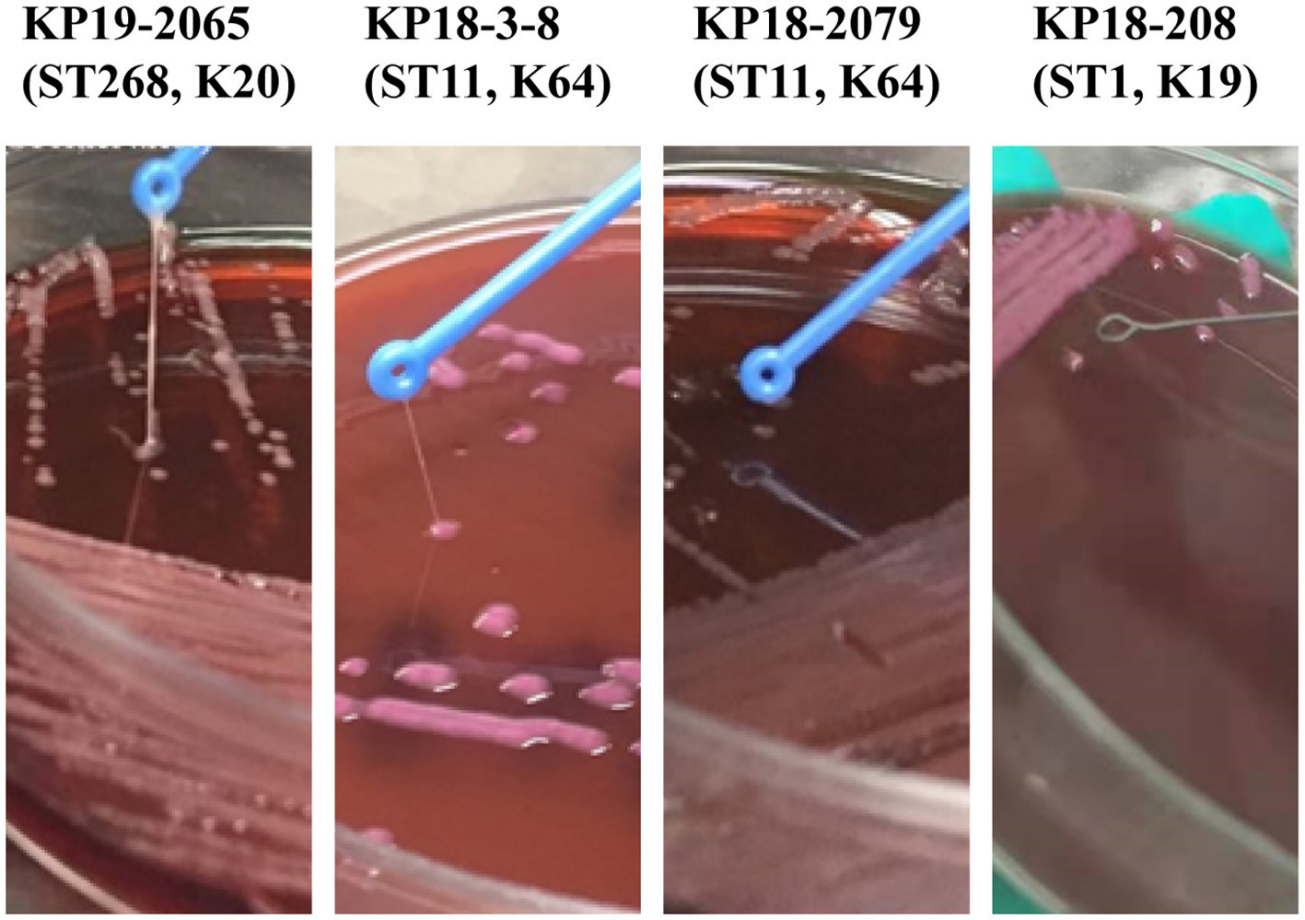
A comparison of *Klebsiella pneumoniae* KP18-2079 and KP18-3-8 with hvKp KP19-2065 and the non-mucoviscous KP18-208. The hypermucoviscosity phenotype of *K. pneumoniae* KP18-2079 and KP18-3-8 evidenced by the string test. After the overnight incubation at 37°C on blood agar, a mucoviscous string of at least 5 mm in length was observed when lifting the KP18-2079 and KP18-3-8 colony using a disposable inoculation loop. The ST268-K20 hvKP strain KP19-2065 was used as a positive control while the non-mucoviscous ST1-K19 strain KP18-208 was used as a negative control.

The mouse infection model was employed to assess the virulence of KP18-3-8 and KP18-2079. As shown in Figure 4, infections of mice with 5 × 10^6^ CFU of strains KP19-2065, KP18-3-8, and KP18-2079 led to a 100% mortality rate at 12 h, whereas the mortality rate was recorded as 0% for the strain KP18-208, a classic CRKP control strain (Figure 4A). Consistently, when infected at the lower dose of 5 × 10^5^ CFU of KP19-2065, KP18-3-8, and KP18-2079, it resulted in a 100% mortality rate at 12, 36, and 60 h, respectively, whereas a 0% mortality rate was observed for KP18-208 (Figure 4B). These data confirmed that KP18-3-8 and KP18-2079 exhibited a virulence level slightly lower than that of KP19-2065 but much higher than that of KP18-208, a classic CRKP strain without a virulence plasmid. In addition, the virulence level of KP18-2079 without the hypermucoviscosity phenotype was lower than that of KP18-3-8, a hypermucoviscous strain which was positive for the string test (Figure 5).

ST11-KL64 CR-HvKP, which was found to be derived from an ST11-KL47–like ancestor through recombination, has demonstrated enhanced virulence and higher mortality rates than that of ST11-KL47 CRKP (Zhou et al., 2020). Moreover, a recent surveillance study on CR-HvKP revealed that ST11-KL64 CR-HvKP became the most common detected CR-HvKP type in different regions of China, especially in Henan province (Zhang et al., 2020). In our study, we isolated two ST11-KL64 CRKP strains that exhibited an XDR profile, with susceptibility only to colistin. Notably, these strains demonstrated heightened virulence compared to their non-virulent plasmid-bearing CRKP counterparts. The presence of composite plasmids encoding both virulence and resistance factors significantly contributed to their carbapenem resistance/MDR profiles and virulent phenotypes.

## Conclusion

Overall, our study identified two novel hybrid virulence plasmids in ST11-KL64 CRKP strains. The IncHI1B/repB-type plasmid pKP1838-KPC-vir co-harboring *bla*_KPC−2_ and virulence factors may have been generated as a consequence of integration of *bla*_KPC−2_-bearing fragment into a typical pLVPK-like virulence plasmid, and the IncFII/IncR-type virulence plasmid pKP18-2079-vir may have been generated as a result of recombination between a typical pLVPK-like virulence plasmid and an MDR plasmid. Our findings revealed further evolution of the pLVPK-like virulence plasmids which were found to be present in ST11 CRKP since 2015 (Gu et al., 2018). The acquisition of such a virulence- and resistance-encoding plasmid, especially the *bla*_KPC−2_-bearing virulence plasmid simultaneously conferring carbapenem resistance and enhanced virulence in classical *K. pneumoniae* isolates, may enable them to become CR-HvKP. The emergence of such strains, which may cause severe infections in individuals difficult to treat with current antibiotics, warrants worldwide attention. Close surveillance on the epidemiology of such hybrid plasmids is urgently warranted to control their further dissemination.

## Data availability statement

The datasets presented in this study can be found in online repositories. The names of the repository/repositories and accession number(s) can be found in the article.

## Ethics statement

The animal study was approved by the Institutional Animal Care and Use Committee of Zhengzhou University. The study was conducted in accordance with the local legislation and institutional requirements.

## Author contributions

FZ: Writing – review & editing, Writing – original draft, Resources, Data curation. LL: Writing – original draft, Formal analysis, Data curation. YZ: Writing – original draft, Formal analysis, Data curation. HD: Writing – original draft, Software, Formal analysis, Data curation. BZ: Writing – original draft, Investigation. XZ: Writing – original draft, Methodology. ZX: Writing – original draft, Data curation. LC: Writing – original draft, Data curation. YW: Writing – original draft, Data curation. RL: Writing – review & editing, Funding acquisition. SQ: Writing – review & editing, Funding acquisition. XF: Writing – review & editing.
